# Surgery, Identity and Embodied Emotion: John Bell, James Gregory and the Edinburgh ‘Medical War’

**DOI:** 10.1111/1468-229X.12720

**Published:** 2018-12-14

**Authors:** MICHAEL BROWN

**Affiliations:** ^1^ University of Roehampton

## I

In 1792, James Gregory (1753–1821), Professor of the Practice of Physic at the University of Edinburgh, became involved in a highly public dispute with a family of man midwives. The row centred on a text attributed to ‘J. Johnson Esq’ entitled *A Guide for Gentlemen Studying Medicine at the University of Edinburgh* (1792). According to Gregory, this was ‘a knavish pamphlet, published under a false name . . . intended to promote the pecuniary interest of one man, and to injure the fame and fortune of another, both of them my colleagues in this University’.^1^ Gregory suspected the author to be the Professor of Midwifery, Alexander Hamilton (bap. 1739, d. 1802), and although the Senate of the university cleared Alexander of any wrongdoing, Gregory's attention then turned to his son, James Hamilton (1767–1839). According to the established account of events, in 1793 Gregory came across James Hamilton in the precincts of the university and, after a brief exchange of words, thrashed him with his cane. Gregory was subsequently called before the Commissary Court where he was ordered to pay £100 in damages, as well as a fine and expenses. Unrepentant, he claimed that he would gladly pay another £100 for the pleasure of doing it again.^2^

As this episode suggests, James Gregory was not averse to engaging in public quarrels with his medical colleagues. Even in the highly fractious world of medicine in late Georgian Edinburgh, he was notorious for his belligerence. Henry Cockburn (1779–1854), the celebrated Whig jurist, claimed that, whatever his virtues, ‘a disposition towards personal attack was his besetting sin’.^3^ In 1809, Gregory was even suspended from the Royal College of Physicians of Edinburgh for refusing to apologize for publishing an account of its internal proceedings.^4^ However, of all the conflicts in which he was involved, none was more protracted, more heated, nor, indeed, more historically significant, than the ‘medical war’ between himself and the surgeon‐anatomist John Bell (1763–1820), which began in 1800 and lasted for up to ten years. This row, which stemmed from a plan to alter the mode of attendance at the Royal Infirmary for members of the Royal College of Surgeons of Edinburgh, played out across the world of print and gave rise to over a thousand pages of ill‐tempered screed.

To date, most scholarly accounts of the life and works of John Bell have tended to view him primarily as the erstwhile mentor of his more famous younger brother, Charles Bell (1774–1842).^5^ Thus, while a number of historians have referred to the ‘war’ between Gregory and Bell, most have done so only briefly, either as context for Charles's subsequent career, or as part of a general account of medical politics in Edinburgh at the turn of the nineteenth century.^6^ The only work that appears to have dealt with the dispute in any detail is an unpublished paper by Michael Barfoot. Indeed, in their reference to the matter, Christopher Lawrence, Stephen Jacyna and Guenter Risse all take Barfoot as their primary authority.^7^ There is now no conceivable way of recovering the substance of Barfoot's argument. However, in his indispensable essay ‘The Edinburgh Medical School and the End of the “Old Thing” 1790–1830’ (1988), Lawrence offers a suggestive insight, stating that Barfoot saw Bell's writings as ‘an attack on elitism and patronage and a defence of equality in the medical world, and Gregory's the reverse’.^8^ Risse also develops Barfoot's observations, arguing that this dispute ‘revealed the critical importance of access to the now famous Infirmary for the fortunes of Edinburgh medicine and surgery – the former basking in worldwide recognition, the latter still mired in mediocrity but strenuously aiming to improve its image and scope of practice’.^9^

For the most part, then, historians have understood this episode in terms of intra‐professional and institutional politics. Quite right too; as we shall see, the Gregory–Bell dispute spans three distinct yet interrelated historiographical fields: firstly, the political culture of medicine in Edinburgh during this period; secondly, the relations between physicians and surgeons at a time when the latter were growing in number and increasingly asserting their status; and thirdly, as I have explored elsewhere, the contested politics of charitable medical institutions in the early nineteenth century.^10^

However, one aspect of the dispute which has yet to be considered is its longer‐term implications for surgery and surgical identity. Gregory's published contributions to the row have a legalistic quality. His 513‐page *Additional Memorial to the Managers of the Royal Infirmary* (1803), for example, exhibits a forensic attention to the minutiae of Bell's writings which more than borders on the tedious. By contrast, though Bell was praised by Gregory for his ‘extraordinary talents’ as an ‘Advocate’ for his fellow surgeons, his publications possess a superior literary quality, so that even Gregory admitted that reading them was ‘an agreeable amusement’.^11^ More than this, in contrast to Gregory, Bell ranged widely. Rather than a mere statement of his position, and that of his fellow surgeons, on the important, yet inherently parochial, matter of access to the Infirmary, Bell's writings developed into an expansive yet coherent statement of surgical identity and ideology. This culminated in his publication of *Letters on Professional Character and Manners: On the Education of a Surgeon, and the Duties and Qualifications of a Physician: Addressed to James Gregory* (1810), a work which not only set out his vision of surgical identity, but did so in explicit contrast to that of the physician. Furthermore, it was against the backdrop of this conflict that Bell produced his magnum opus, *The Principles of Surgery* (1801–6), a work that would continue to be read as a standard text for decades to come.

This dispute, and John Bell's contribution to it, is thus highly worthy of scholarly analysis, not only because he was regarded as ‘the only true surgeon in Edinburgh’^12^ and ‘the best surgeon that Scotland had then produced’,^13^ but also because, some thirty years after their publication, Bell's writings were still functioning as a touchstone for surgical identity, or what were then called ‘surgical morals’.^14^ Indeed, it would not be unreasonable to suggest that Bell was one of the most important influences on surgical culture in Britain in the first half of the nineteenth century.

This article contends that what made Bell's work so appealing to large numbers of surgeons in the late Georgian period was its articulation of a culturally resonant professional identity shaped, to a profound degree, by *feeling* and *emotion*. As Thomas Dixon has argued, the term ‘emotion’ has a complex history. In the eighteenth century it functioned ‘either as an undefined and general term for any kind of mental feeling or agitation, or sometimes as a stylistic variant for central theoretical terms such as “passion” and “affection”’.^15^ Dixon regards Charles Bell as the ‘coinventor’, along with Thomas Brown (1778–1820), of the ‘modern’ concept of emotion.^16^ However, as with so much of Charles Bell's work, it is clear that, whatever the difficulties of their relationship, he owed much to the influence of his older brother and mentor, John. As we shall see, emotion saturates Bell's writing and underpins his conception and idealization of surgery and the surgeon, yet it has passed largely without comment, certainly without analysis. To be sure, some of his biographers have suggested that Bell was a man who united ‘compassion’ with ‘moral courage’ or that he ‘was a kind, generous, and compassionate man’, but none have sought to interrogate the role of emotional expression in shaping his public identity, nor have they sought to situate his surgical persona within a broader social, cultural and political context.^17^

The history of emotion is now a well‐established field of study but it is only in relatively recent years that historians of medicine and science have begun to take emotions seriously in their consideration of professional practice, identity and representation.^18^ As we have seen, historians of medicine have long been sensitive to the political dynamics of professional relations. Historians of emotion have likewise drawn attention to the politics of feeling, while literary scholars and cultural historians have scrutinized the politics of sensibility in the later eighteenth and early nineteenth centuries.^19^ However, these bodies of scholarship have rarely spoken to one another. What this article seeks to do, therefore, is to excavate the cultural and political meanings of the Gregory–Bell dispute through an analytically inclusive approach. It will demonstrate that this dispute can best be understood by mapping the rivalry between physicians and surgeons in late Georgian Edinburgh onto emergent fault lines within the predominant ‘emotional regime’ of the period. Bell's construction of the surgeon as man of feeling, it argues, tapped into wider debates about sincerity, sentiment and sensibility that first took shape in the 1780s and which came to the fore in the fraught, post‐Revolutionary years of the 1790s. In these debates, the tropes of sensibility were themselves deployed as a critique of affected, theatrical or disingenuous emotional expression of a kind that threatened to undermine the credibility of authentic experience and sensation.^20^

In the context of the Gregory–Bell dispute, this critique of affectation centred on the meanings assigned to embodied knowledge and practice. In his work on the history of emotion as a concept, Thomas Dixon mistakenly refers to Charles Bell as a physician, rather than a surgeon. This is intriguing, because those embodied, physiological qualities, such as the motion of the heart and lungs, which Bell brought to the ‘modern’ concept of the emotions, were a direct product of his surgical world‐view.^21^ In this sense, too, we can see a prefiguration in the work of his older brother. For John Bell, this article suggests, sincere emotions were rooted in embodied experience. Traditionally, the social status of the physician, though by no means assured, was sustained by his relative distance from manual work. By contrast, the surgeon remained inextricably associated with praxis and craft skill. Gregory played on this distinction, expressing his distaste for surgical operations and anatomical dissection and repeatedly alluding to surgery's roots in trade. Bell, on the other hand, sought to turn such prejudices on their head, asserting that it was precisely through embodied experience and an exposure to the physical suffering and mental distress of his patients that the surgeon came to be formed as a man of honest, heartfelt sensibility, an emotional sincerity that he situated in marked contrast to the ornamental manners of the physician, who was professionally isolated from such visceral experiences. This had profound social and political connotations. As Barfoot has suggested, and as we shall explore in more detail, Gregory's conception of medicine and its relation to surgery was rooted in a fundamentally aristocratic conception of social worth. Bell's representation of surgery, by contrast, whatever his actual political views, resonated with a more democratic and reformist vision in which the ‘manly simplicity’ of the aspirant professional stood in opposition to the lordly affectations and dissimulation of the established elites.

## II

The medical world of late eighteenth‐ and early nineteenth‐century Edinburgh has been so well documented by historians that little more needs to be said here, save as context.^22^ What is important to note, is that the highly factious nature of the Edinburgh medical community owed much to the presence of the university medical school which, controlled as it was by the town council, was immersed in local civic politics.^23^ The medical school had been formally established in 1726 by the Lord Provost George Drummond (1687–1766) and the surgeon John Monro (bap. 1670, d. 1740) with the intention of imitating the medical teaching of Herman Boerhaave (1668–1738), under whom Monro had studied at the University of Leiden. Although it lost its distinctly Boerhaavian flavour within a generation, the Edinburgh medical school continued to provide a systematic and comprehensive education which distinguished it from other universities in the British Isles. Its ‘Golden Age’ was marked by the tenure of William Cullen (1710–90), first as Professor of Chemistry (1755–66) and subsequently of the Institutes (theory) (1766–73) and Practice of Medicine (1773–89).^24^ Other luminaries of the era included Joseph Black (1728–99), Alexander Monro secundus (1733–1817) and John Gregory (1724–73).

One of the singular features of Edinburgh medical education was clinical instruction, a model of teaching imported from Leiden. To that end, a small six‐bedroom infirmary was opened in 1729 followed in 1741 by a much larger purpose‐built structure, now known as the Royal Infirmary, in Thomson's Yards.^25^ The situation of this building, almost exactly halfway between the College and Surgeon's Hall, served as a neat metaphor for the contested politics of its foundation. In what would appear to have been an attempt by Alexander Monro primus (1697–1767) to monopolize the surgical department of the Infirmary, the Charter of 1736 stated that the patients would be ‘entertained and taken care of by the Royal College of Physicians and *some* of the most skilful surgeons’.^26^ Slighted by their effective exclusion from the Infirmary, the Incorporation of Surgeons established their own hospital in the same year. However, under the leadership of Deacon John Kennedy, they soon approached the managers of the Infirmary and offered to merge the two institutions, promising to transfer all subscriptions and pay some £500 in return for the members of the Incorporation being allowed to attend the Infirmary by right; this offer was accepted in 1738.^27^

It is important to note the wording employed by the Incorporation in their appeal to the managers; they claimed that the system of universal attendance was necessary ‘in order to preserve an equality among the surgeons of Edinburgh’.^28^ For its opponents, this language was suggestive of the Incorporation's guild mentality, but it might just as well be interpreted as a collective desire on the part of surgeons to defend their interests against the dominance of the Royal College of Physicians. By this time the Incorporation of Surgeons was in a somewhat anomalous position. In 1722 the surgeons had split from the barbers (twenty‐three years before their London colleagues) but they retained strong ties to the town council and would keep formal membership of the Edinburgh Trades long after the Charter of 1778 had made them the Royal College of Surgeons. While this association with the Council was useful, given the hegemonic influence it exercised over civic affairs, it was also a potential impediment to their desire to be regarded as learned gentlemen, rather than tradesmen.

Although formal membership of the Incorporation/Royal College fluctuated across the century, by the later 1700s the number of surgeons in Edinburgh was increasing. More importantly, perhaps, surgical knowledge was also on the rise. During the long years of war with France (1793–1815), there was increased demand from the army and navy for medical practitioners. What each regiment or ship‐of‐the‐line needed was not a physician but a surgeon, or rather surgeon‐apothecary, capable both of performing operations and of attending to the quotidian medical needs of the men.^29^ As Lawrence has suggested, the medical school could not, and did not, respond to this state of affairs. For one thing, the Professor of Anatomy, Alexander Monro tertius (1773–1859) was a physician and taught anatomy in a deeply traditional manner which focused on form and function. By contrast, a number of lecturers in Edinburgh were offering extramural courses which viewed the body through the eyes of the surgeon and which catered to his needs.^30^ It has been suggested that Charles Bell was the originator of surgical anatomy in Britain but, once again, it is clear that he owed much to his brother.^31^ John Bell was one of the most popular extramural lecturers in the city and, as we have heard, was considered to be ‘the only true surgeon in Edinburgh’^32^ in that he taught surgery as an applied *science*.^33^ ‘ANATOMY’, he claimed, ‘serves to a Surgeon, as the sole theory of his profession, and guides him in all the practice of his art.’^34^

Despite his eminence, Bell was not without his critics. Nor were the surgeons of Edinburgh a united body. Indeed, the period immediately preceding the dispute between Bell and Gregory was characterized by the latter as one of internecine ‘chirurgical warfare’.^35^ In 1799, an unknown author, possibly either John Barclay (1758–1826), Bell's former assistant, or John Thomson (1765–1846), perhaps Bell's greatest professional rival, published a pamphlet under the pseudonym ‘Jonathan Dawplucker’ entitled *Remarks on Mr John Bell's Anatomy of the Heart and Arteries*.^36^ This squib was a rather limp attack on Bell's writing, but he nonetheless took great offence, not so much at its content, as at the way in which it was ‘stuck up like a Play‐Bill in a most conspicuous and unusual manner, on every corner of this city; on the door of my Lecture‐room! on the Gates of the College, where my pupils could not but pass! and on the gates of the Infirmary, where I went to perform my operations’.^37^ In response, Bell published his own pamphlet in the character of Dawplucker attacking the *System of Surgery* (1783–8) of another (unrelated) surgical rival, Benjamin Bell (1749–1806).^38^ By comparison with the first Dawplucker pamphlet, this was a highly accomplished literary excoriation which castigated Benjamin Bell for his impetuous and unscientific approach to surgery. For our purposes, what is especially notable is the way in which it presented Benjamin Bell as a man consumed by feelings of anger and fear rather than confidence and compassion:

The difference betwixt your description and that of a bold operator, is just that which distinguishes an assassin from a brave man! You write bloodily, though not boldly: you speak not like a regular surgeon . . . but like a desperate man, careless of everything, and afraid only of being affronted, or, in other words, ‘embarrassed’ in the midst of a public exhibition! You write like one who had been often caught and entangled in difficulties from which he had no other way of disengaging himself than by a slap‐dash stroke of the knife . . . You are enfuriated [sic] by opposition! the words adhesion, stricture, gut, and sac, excite proportioned fury! and you exclaim, tear, cut, clip, destroy – Tear the adhesions, cut every thing; – surgery consists in cutting! and the best surgery is to cut every thing!!!^39^

Quite how Bell came to be involved in this dispute is not entirely clear. The evidence suggests that he was a ‘pugnacious’ and quarrelsome man; ‘No formidable insect delighted in his sting so much as he did’, wrote Cockburn.^40^ However, there may also have been a political dimension. As Lawrence has stated of Edinburgh, ‘the French wars saw the apogee of the ascendancy of Henry Dundas, and the almost total support by the traditional elite for Tory policies . . . Scots Whigs were in a minority both in parliament and in Scotland’.^41^ And yet a Whig faction was making increasing inroads into the city and university, leading to conflict over much‐coveted public offices.^42^ Charles Bell has been described by some historians as a conservative Whig, but, within the particular context of Scottish politics, John's inclinations are not quite so clear cut.^43^ The fact that his father, Rev. William Bell (1704–79), left the Church of Scotland for the Episcopal Church at a time when it was indelibly associated with Jacobitism suggests Tory sympathies,^44^ while John's membership of the Highland Society of Scotland points in a similar direction.^45^ And it is perhaps no coincidence that his key surgical opponents included such prominent Whigs as John Thomson and John Allen.^46^ However, as Lawrence has pointed out, the medical politics of Edinburgh in this period cannot always be conveniently mapped onto party politics and, as the example of Bell's contemporary, Robert Burns (1759–96), demonstrates, disentangling Jacobitism from broader political principles can be tricky.^47^ Certainly, Bell's possible Toryism does not sit comfortably with his seemingly democratic take on medicine and surgery.

## III

If being the son of an impoverished and marginalized clergyman made John Bell something of an outsider, then James Gregory was the quintessential insider. He was born in 1753 to John Gregory, physician and sometime lecturer in philosophy at King's College, Aberdeen. His father was appointed Professor of the Practice of Physic at Edinburgh in 1766, becoming celebrated for both his medical and moral writings.^48^ James Gregory had moved with his father to Edinburgh in 1764, aged eleven, and initially studied for a degree in the arts. After a brief stint at Christ Church, Oxford, he returned to Edinburgh in 1767 to study medicine. During the course of his studies his father died and James took up his post as a temporary professor. He graduated MD in 1774 and, after two years spent studying on the Continent, returned to Edinburgh once more to take up the now vacant post of Professor of the Institutes of Medicine.^49^ He was a mere twenty‐three years old, a shining example of the nepotism for which Scottish universities, and the Edinburgh medical school in particular, were infamous.^50^

It was perhaps the entitlement and security which came from such privileged connections that gave Gregory the confidence to engage in as many feuds as he did. In 1798 he was elected President of the Royal College of Physicians, a post which gave him an automatic seat on the governing council of the Royal Infirmary. By this time the issue of surgical attendance at the Infirmary had still not been resolved. In addition to four salaried posts, created in 1769, the surgeons attended by rotation for a period not normally exceeding two months. However, their subordinate status was evident in the fact they were not allowed to undertake any operation ‘of importance’ without the permission of the physicians‐in‐ordinary and, as a result, many of the more senior surgeons declined to attend.^51^ In an ostensible desire to regularize the system, the year 1800 saw a number of authors propose significant changes to the covenant of 1738. Two of these men, Benjamin Bell and John Thomson, were, as we have seen, opponents of John Bell.^52^ However, it was Gregory, a man who had been on relatively cordial terms with Bell up to this point, whose intervention would initiate a war of words.^53^

This intervention was a tract entitled *Memorial to the Managers of the Royal Infirmary* (1800) in which Gregory proposed the permanent appointment of ‘a number of ordinary attending Surgeons’ as well as ‘a regular supply of younger Surgeons’ and a ‘sufficient number of *extraordinary* or *consulting* surgeons . . . to give their advice and assistance in all extraordinary and difficult cases’.^54^ Gregory's suggestions were little different from those of Thomson and Benjamin Bell. What was remarkable was the general tenor of his argument; whereas those two authors approached the issue as surgeons, Gregory did so from the standpoint of the pre‐eminent physician in Edinburgh. Referring back to the Charter of 1736, he suggested that its stipulation that patients be attended by ‘*some of the most skilful Chirurgeons*’ was significant because:

It implies, that, of the Surgeons in Edinburgh, some may be *more* and others *less* skilful. This, I presume, many people would very readily believe without the evidence, either of a ghost or of a Royal Charter . . . it is generally known to be the case with the individuals of many different professions; very remarkably among lawyers and wig‐makers, shoemakers and tailors, milliners, cooks, fiddlers, dancing‐masters, postilions, and physicians.^55^

Several tropes that would become recurrent themes of Gregory's argument are worth noting here: firstly, the reference to ‘a ghost’, an allusion to the fatal consequences of surgical incompetence; secondly, the rhetorically expedient lumping together of high‐status ‘professions’, such as law, with lower status trades such as cookery; and thirdly, the disingenuousness of including physicians in this list. After all, the Charter granted the right to attend the Infirmary to *all* members of the Royal College of Physicians, not ‘some of the most skilful’. That proviso was reserved for surgeons.

This invidious distinction between individual competence and collective authority is essential for understanding both Gregory's argument and the reaction to it. While he repeatedly claimed that the ‘merits or demerits of any individual’ were ‘unnecessary for the object which I have in view’, his refusal to single out individuals meant that his criticisms necessarily fell upon the surgeons as a body. In particular, they fell upon the mostly junior members of the College of Surgeons who, unlike many of their senior brethren, ‘rigorously avail[ed] themselves’ of their right of attendance at the Infirmary. The practice of surgery in the Infirmary was, Gregory claimed, ‘*entirely* in the hands of the youngest and most inexperienced surgeons’.^56^

Despite his refusal to name names, Gregory peppered his text with sensational examples of surgical incompetence, particularly in regard to manual dexterity, a quality which, he claimed, could only be gained by experience.^57^ In perhaps the most notable instance, he referred to a surgeon ‘whose hand shook so much’ that ‘he should scarce have undertaken to apply a plaster or a bandage’, yet who regularly performed operations at the Infirmary, including a delicate operation on the eye. In this case, Gregory's informants were the students of medicine who attended the hospital. Even he was forced to admit that ‘it may appear worse than strange, perhaps reprehensible . . . to refer with any kind of respect to the opinion of the students on this subject; which opinion they used sometimes to express in a most indecent manner, by murmuring or even hissing in the operation‐room’. And yet, by doing so, Gregory effected a remarkable inversion of status and authority, the qualified surgeons of the Infirmary being held accountable to the judgement of even the most junior medical student.^58^

In his criticism of the system of surgical rotation, Gregory resorted not only to sensationalism but also to moral outrage. Writing in the languages of Christian charity and paternalism, given particular inflection by the cultures of sensibility and humanitarianism, he stated:

The poorest patients in the Hospital have the understanding, the feelings, and the rights of men. They know and feel, though they may not have the words to express it, that misery is sacred. They are *our* families, and *our* children, when we act as Managers of the Hospital: They have a right to our paternal care; they have a right to obtain from us the best assistance, and every relief and comfort which *we can* procure for them.^59^

Therefore:

Whatever is learned; or whatever real or supposed improvement is acquired at the *expense* of the poor patients, I mean by any *unnecessary suffering*, or *danger*, or *harm* to them, is *injustice* and *cruelty* instead of *charity*, *benevolence*, and *brotherly love* to the sufferers; it is a breach of trust on those who do it, or permit it; it is an outrage on human nature.^60^

While Gregory claimed that it was ‘not the *fault* of a youth of four‐and‐twenty, just going to begin the practice of his profession, that he has not the skill, and useful experience, and manual dexterity of a surgeon fifty years of age’, he clearly thought it *was* the fault of surgeons as a corporate body. Indeed, he repeatedly claimed that the agreement made by Kennedy and the Incorporation of Surgeons with the managers of the Infirmary in 1738 was a wilful act of self‐interested cruelty: ‘I doubt’, he wrote, ‘whether there was ever in this world, or ever can be, a more direct, avowed, and disgraceful opposition between the interests, real or supposed, of a corporation, and the interests and the rights of humanity.’^61^

Gregory could have closed his argument there, but he did not. Rather than limiting himself to the issue of surgical rotation at the Infirmary, he proceeded to critique surgery as a whole and to question its place within the social landscape and political economy of the city. The Incorporation's stated desire to ‘preserve an equality among the surgeons of Edinburgh’ was anathema to Gregory. The reason why they were so keen to preserve ‘equality’, he claimed, was because there was simply too many of them. A certain level of competition was necessary to ensure a tolerable standard of competence, perhaps twenty physicians and twenty surgeons in a city where only ten of each were really needed:

But it would be no advantage to the public, any more than to the Faculty, to have *two hundred*, or *one hundred*, or even *forty* of them established in such a town . . . Physicians and Surgeons are by no means on the same footing, in point of political economy, with corn and cattle; a superfluity of which, in the course of nature, is soon converted into an additional number of men and women, to the most essential benefit of the state. They cannot even be applied to various uses, like a superfluous quantity of wool, and flax, and iron, and other raw materials used in manufactures; nor can they be exported and bartered by way of trade for other valuable articles, like a super‐abundance of highly‐manufactured goods, beyond what their own country can consume. The superfluous physicians and surgeons are absolutely useless and helpless . . .^62^

Gregory's awkward fusion of the political economics of Adam Smith (bap. 1723, d. 1790) with the mercantilism it would displace is indicative of his innate conservatism in the face of profound social, economic and intellectual change. Gregory was a man of the *ancien régime*, an aristocrat in outlook, if not in law. Just as his conception of charity was rooted in a paternalistic benevolence which was being challenged by the twin forces of political economics and utilitarianism, his understanding of society was similarly shaped by the face‐to‐face relations of the pre‐modern world, rather than the abstractions of social and cultural modernity.^63^ Hence, his estimation of the appropriate number of medical practitioners for the city of Edinburgh was predicated less on an objective assessment of economic *demand* than a subjective assessment of social *worth*. Deprecating the notion of surgical ‘equality’, he claimed that he preferred ‘inequality’ and looked with barely concealed disdain on the growing ranks of surgeon‐apothecaries emerging from the private anatomy schools:

Their number would soon increase rapidly, by the addition of all who had the requisite qualifications, and so little activity, or spirit, or honourable ambition, as to be contented for life with the miserable pittance and degrading situation which such a system of equality would insure to them. Surgery in Edinburgh would soon cease to be a learned or a liberal profession. Those who practised it would not be ranked in public estimation with physicians, or merchants, or lawyers; nor would any of them be known and esteemed as authors of learned, scientific, and useful works on anatomy, chemistry, physic, or surgery . . . In one short sentence, by such a system of equality, the Surgeons of Edinburgh would soon become Barbers again.^64^

Gregory did not mind surgeons *per se*. He counted a number among his close friends. But while he admired a talented few, the rest he dismissed as ‘arrant Drones’.^65^ For Gregory, authority did not derive from qualifications or even from office, but rather from character. Referring to the idea that all medical practitioners were more or less (in)competent, he contrasted the individualized nature of medical services with the anonymity and interchangeability of the urban worker:

No person can be so stupid as . . . to wish that a perfect equality were established among physicians and surgeons respectively, so that in case of need, he might call a physician or a surgeon, without knowing his character, or even his name; just as he might call a porter or hackney coach, without enquiring the name of the porter or the character of the coachman.^66^

For Gregory, the true signifier of character and, hence, social worth was wealth. In this sense, the surgeons of Edinburgh were in a highly precarious position. A minority, he suggested, ‘are perpetually rolling about the streets on four wheels, while three or four times as many of them walk about the streets on their two hinder legs in true primitive simplicity’. And while ‘it is of little or no moment to [the patient] whether the operator come to his house on foot or in a gilded chariot . . . I should rather think it some comfort . . . in those anxious and fearful moments, to know that his surgeon has such an extensive and successful practice that he can afford to keep such a chariot’. Indeed, if the surgeons were inclined to pursue equality, Gregory argued, ‘In a few years, not one of them would be able to live in a genteel, or what at present they think a decent and comfortable manner, nor would any of them be admitted into the company of gentlemen.’^67^

As is clear, Gregory repeatedly goaded surgeons about their origins in trade and suggested the possibility, even likelihood, of social regression. But, just as importantly, he played upon another well‐established stigma associated with surgery: cruelty. More than simply drawing attention to what he perceived to be the cruelty of allowing inexperienced surgeons to attend the Infirmary, he went so far as to imply that surgery as a whole was inherently cruel. Drawing upon an appropriate metaphor for the period, he claimed that:

Many of our greatest heroes, both in red coats and in blue, men who would boldly march up against a battery of cannon, or joyfully obey an Admiral's signal for close action and breaking the line of battle of an enemy's fleet, will grow pale at the sight of only two or three Surgeons, when these come to consult about them. And many a poor patient, when he is set down in midst of five and twenty Surgeons in this hospital, I have no doubt, feels all the horror, without the faith of the Prophet Daniel, when first he took his seat in the den of lions.^68^

Such observations about the emotional demands of surgery were, as we shall see, not uncommonly made by surgeons themselves, but coming from a physician, especially one so openly disdainful of surgeons and surgical science, they were doubtless galling. Gregory even wrote that:

I never am present at any operation in private practice, unless at the patient's particular desire; of course I see such operations very seldom. Though I have been intimately connected with the Infirmary as a clinical professor for more than three and twenty years, I have not during all that time been present at a public operation in the Theatre, where the junior Surgeons chiefly operate, but one day.^69^

In a later text, Gregory stated that he ‘neither like[d] the sight nor the smell of a putrid mangled human body’,^70^ and this distaste for the working life of surgeons was similarly evident when he imagined what would happen when his text was published: ‘When this Memorial shall be laid upon the table in Surgeons Hall’, he wrote, ‘five and forty scalpels, sharper than razors, shall be drawn at once to dissect it to the bone.’^71^

## IV

Gregory was correct to assume that his *Memorial* would be dissected by the denizens of Surgeons’ Hall, but he can have had little idea that this task would fall to perhaps the most gifted anatomist in Edinburgh. Bell had formerly expressed himself loath to comment on the issue of surgical attendance at the Infirmary, but he was sufficiently offended by the tone of Gregory's text to agree to a request, made by the junior members of the College of Surgeons, that he publish a response.^72^ In the resulting *Answer for the Junior Members of the Royal College of Surgeons of Edinburgh to the Memorial of Dr James Gregory* (1800), Bell claimed that the surgeons were a ‘public body’ who ‘demand respect’. Instead, their profession had been ‘cruelly traduced’. ‘He mocks at all dignity’, Bell claimed, ‘at all semblance of science, at all professional skill, faith, honesty, or honour; and we and our cruelties are his constant theme.’^73^

This emphasis on cruelty is revealing. Bell was particularly sensitive to the charge that surgery was, in his words, ‘a cruel trade’.^74^ As such, it is of little surprise that he should seek to defend surgeons’ respectability, both social and moral. What is more surprising, perhaps, is the relative weight that Bell gave to the latter. Indeed, compared to Gregory's incendiary yet relatively infrequent suggestions of surgical cruelty, Bell's appeal to compassion, sympathy, and emotion in general, is quite remarkable; it saturates his text, functioning as its central motif. For example, he wrote that:

To become skilled [in surgery], a man must live among the sick: he must have lively feelings, and a sympathizing nature; his mind and senses must be deeply impressed with the character of every kind of suffering; he must have that inward sympathy with the distresses of his fellow‐creature[s], which fills the mind with sincere and affectionate interest. What can more aggravate sickness, than to tell the long tale of misery to one who merely listens, who betrays no touch of compassion, whose cold and formal inquiries imply no interest, and end with a prescription in form[?] Such a man never learnt his profession, will never learn it: he has no feelings towards his individual patients, and can have no enthusiasm towards his general duty. In our profession, young men should have instilled into their minds that sympathy with the sufferings of their patient, and that keen spirit of investigation should be roused in them, which refines every sense, and quickens the intellect.^75^

Thus, in contrast to Gregory's accusations of cruelty, accusations which tapped into well‐worn negative stereotypes, Bell suggested that the identity of the surgeon was positively predicated on sensibility, on the capacity for compassion and sympathy. These were not simply manners, mere ornamental social graces. Rather, according to Bell, a highly attuned sensitivity to the feelings of one's patients was a vital clinical skill, and any surgeon who did not possess ‘lively feelings, and a sympathizing nature’ had not ‘leant his profession’. Indeed, not only was the surgeon to feel compassion for his patients, he was also to become what I have called elsewhere a kind of ‘emotional savant’, able to read the most subtle expressions of feeling in others and act with calming reassurance.^76^ Bell reiterated the point:

To be initiated into our profession, is not merely to be taught the principles of Chemistry, and the Anatomy of the human body; but it is . . . to feel an interest in the fate of each patient; to form apprehensions for his safety which perhaps he himself does not feel . . . to be alarmed by changes of voice, pulse, and countenance, which make no impression even on the patient's friends. This is the true initiation in to our profession; and he, who is once full of these sympathies, takes an interest in every case, and studies with unremitting diligence.^77^

In presenting sensibility as an integral part of surgical practice and identity, Bell sought not merely to deflect accusations of cruelty, but also to question Gregory's own public identity. As we have seen, Gregory's *Memorial* drew heavily upon the same rhetoric of Christian charity and sensibility that had been deployed by his father. However, Bell questioned the sincerity of such expressions, especially when compared to the embodied experiences of the surgeon:

Has his mind been thus keenly touched, almost disordered, at the miseries of his fellow creatures? No, no! his strong sensibilities we hold but lightly: He never passed a sleepless night, reflecting what was to be done on the morrow; never witnessed the severities of the surgeon; never strained hard his breath, nor involuntarily clenched his hands at the sight of another's agony; nor blanched with fear, nor felt the palpitations of anxiety, in the midst of an eventful operation? Let a man feel the things he can feel, and his sensibilities will be applauded. This sensibility is not of the right stamp: he coolly collects his jests, when he would be witty; and as coolly strains out a lamentation when he would be thought humane.^78^

Bell's use of the phrase ‘right stamp’ is telling. In this period a stamp might suggest the mark made upon goods on which duty had been paid, something which, whatever its formal purpose, was often construed, by manufacturer and public alike, as a signifier of authenticity.^79^ Bell was therefore suggesting that Gregory's sensibility was somehow inauthentic or insincere. Rather than truly feeling, as the surgeon did, Gregory merely deployed sensibility as a rhetorical device, ‘coolly’ affecting a lamentation so that he might appear ‘humane’.

Bell's critique of Gregory's emotional affectations was rooted in wider debates about the merits of sensibility. As Markman Ellis has argued, while sensibility lacked ideological coherence, its capaciousness and elasticity only served to enhance its cultural appeal across the eighteenth century.^80^ Its origins were eclectic, ranging from Latitudinarian theology and moral philosophy to nervous physiology; so too were its manifestations, readings and applications. Nevertheless, it might best be defined as an intellectual and cultural phenomenon which encouraged an openness to feeling and which saw in the capacity for sympathizing with others a means for improving social relations. Edinburgh was central to the elaboration of sensibility; its philosophical lynchpins, David Hume (1711–76) and Adam Smith, were towering figures of the Scottish Enlightenment, while it was the nervous theory of Edinburgh physicians Robert Whytt (1714–66) and Monro secundus that made sympathy a physiological as much as a moral principle.^81^ Moreover, it was in Edinburgh that sensibility is often held to have reached its apogee, at least in the British Isles. In 1771 Henry Mackenzie (1745–1831), an associate of both Bell and Gregory, published *The Man of Feeling*. Perhaps the quintessential sentimental novel, its protagonist, Harley, embodies an ‘ideal sensibility’, and ‘sheds tears over the misfortunes of the people he meets’.^82^ Though hugely popular in its day, by the early nineteenth century it had fallen from favour, its profuse lachrymation rendering it culturally unpalatable.^83^ The reason for this lay in the fact that, during the 1780s, a number of commentators, such as Vicesimus Knox (1752–1821), had become increasingly ‘anxious about insincere assertions of sensibility’.^84^ The French Revolution of 1789 (and particularly the Terror of 1793–4) also played an important role. The sentimentalism associated with Revolutionary culture, particularly the place of tears as a signifier of political virtue, heightened concerns about the social and moral hazards of ostentatious emotional expression.^85^ However, as Ellis observes, ‘the politicisation of sensibility had largely occurred by the end of the 1780s’ and the anxiety over the emotional dynamics of the Revolution was hence part of a wider ‘sensibility controversy . . . and not just a symptom of it’.^86^

One of the consequences of this ‘politicisation of sensibility’ was that some began to place increasing emphasis on the sincerity and authenticity of emotional experience as opposed to the mannered artifice of social performance. Indeed, as a number of scholars following Lionel Trilling have observed, emotional sincerity and authenticity were among the key markers of an emergent culture of Romanticism, and its peculiar inflection of sensibility.^87^ Bell's *Answer* to Gregory must be understood in this context. As the earlier quotation demonstrates, Bell contrasted the emotional experience of surgery with Gregory's merely rhetorical appeal to feeling. While this might itself be interpreted as a rhetorical device (and of course it was), it is also indicative of Bell's wider conception of surgery. Throughout his oeuvre there is a powerfully embodied quality to the descriptions of operative surgery which transcend the functional requirements of surgical instruction and embrace, instead, the visceral, affective dimensions of surgical experience. Take, for example, the following passage from his *Discourses on the Nature and Cure of Wounds* (1795), in which he describes an operation for the relief of an arterial aneurism:

You are to . . . expect that the moment you cut the tumour, the blood will rush upon you with a terrifying violence: nor should you ever expect to clean the great cavity with sponges or cloths, for the artery will fill the cavity with blood, faster than you can throw it out, till the patient breathes his last. Instead of this, you draw the knife deliberately and fairly over the tumour, so as to lay it open. The skin being thus divided, the great livid bag of the aneurism surrounded with its strong fascia, rises into view. Next you push your lancet into the bag, and then do all that remains in your operation with great boldness; run your bistory upwards and downwards so as to slit up the tumour quickly; plunge your hand suddenly down towards the bottom; turn out the great clots of blood with your hand and fingers, till having reached the bottom entirely, you begin to feel the warm jet of blood, and directed by that, clap your finger upon the wounded point of the artery, as it is but a point, your finger will cover it fairly, and your feeling the beating of the artery, assures you that all is now safe.^88^

For Bell it was precisely the embodied nature of his work, and his exposure to the extremes of human pain, suffering, and endurance, which rendered the surgeon uniquely sympathetic and emotionally astute. ‘Wherever we turn, it is towards objects of compassion’, he wrote in his *Answer* to Gregory:

We pretend no finer feelings than nature has implanted in every common breast ‘but we are accustomed to see people, once the gay and the happy, sunk in deep, retired distress: sometimes devoted to a certain, but painful and lingering death; sometimes struggling with bodily anguish, or the still fiercer tortures of the mind’. We venerate the miseries of our fellow creatures, and the profession that brings relief.^89^

As we have seen, Gregory expressed what Bell referred to as ‘a perfect horror . . . at the unnecessary presence of surgeons’ and claimed to have not attended an operation in over twenty years of practice.^90^ Indeed, in his *Additional Memorial* Gregory reiterated his distaste for surgery and ridiculed Bell's description of the affective qualities of surgical experience, writing ‘Whether I am or am not subject to hysterics, and accustomed to exhibit the horrid grimaces thus beautifully described by Mr John Bell, is a question of no moment.’^91^ For Bell, however, Gregory's absence from the emotional space of the operating theatre rendered his profession of feeling inherently insincere:

Had you but called your metaphysics to your aid, and trusted rather to your reason than your feelings, you might have conjectured, that in this simple description all must be right; that what you imagined to be merely cunning, was but a simple unostentatious description of emotions very natural to those not altogether void of humanity. I do indeed believe, Sir, that if you ever again pretend to express excessive sensibility, it will be . . . ‘in HORRIBLE GRIMACES;’ and it would no doubt be painful to see you disordered by a feeling, or the affectation of a feeling, as foreign to your physical and spiritual frame, as ‘Hysterics’ are to robusteous [sic] health. Be advised, Sir, and write no more on sensibility and charity!^92^

What is remarkable about this aspect of Bell's argument is that it turned conventional professional and social prejudices on their head. Gregory, like many physicians, deprecated surgeons because the manual nature of their work rendered them closer to tradesman and inferior to the intellectual labour of the physician. Traditionally, too, sensibility was held to be a quality of nervous refinement more marked in the gentleman and lady than in the horny‐handed working man or woman. By contrast, what Bell was suggesting was that the embodied emotional experience of surgery had a morally improving quality which was lacking from the conventional practice of the physician. As such, it was the surgeon, rather than his supposed social superior, who was the truly authentic man of feeling, and it was this feeling that gave the surgeon his sense of professional self‐worth. As he later wrote, ‘I have spent much of my life in services which could only arise from those feelings, in useful and active duty, which you would disdain.’^93^

It is not hard to see the political implications of this argument. As we have heard, Gregory's principal objection to the established system of surgical rotation at the Infirmary was that it allowed inexperienced surgeons to practise on poor patients. More generally, Gregory thought that a few genteel surgeons were preferable to an army of low‐status ‘drones’. In opposition to this fundamentally aristocratic understanding of professional and social relations, Bell advanced what we might call a democratic model of surgical identity. This vision was in keeping with an inchoate conception of the medical profession that I have explored elsewhere.^94^ It assumed that the worth of the surgeon was not predicated on a subjective assessment of individual character but was, rather, an objective corollary of office and qualification. According to Bell, all surgeons (or at least all those for whom he imagined himself the representative) were equally qualified and equally worthy, in that they possessed the same qualifications and the same status. Moreover, there was no such thing as an ‘inexperienced’ surgeon because skill was a function of *expertise* derived from a scientific education (of the kind Bell provided) and not a product of mere *habit or routine*:

There is all that difference which reflection should lead us to expect, betwixt a man learning BY EXPERIENCE to become an operator, and one taught BY SCIENCE, and prepared by every study that may contribute to success. The one is supported by conscious knowledge and skill, in a manly, steady, dignified state of mind; the other, fluttering with fear and anxiety, and greedy of applause.^95^

Even if the surgeon derived his authority from scientific training rather than from individual character, this is not to say that character was unimportant. However, this character was not of the kind that Gregory admired, namely the social graces of the gentleman. It was not what Bell dismissed as ‘the mere accomplishments, mere ornaments’ of ‘gaudy trivial beings, all fluttered with metaphysics, philosophy [and] literature’. This was ‘the finery’ with which, according to Bell, Gregory had ‘strutted most insignificantly through life’. Instead, surgical character was of a moral kind, shared by all surgeons. It was simple, honest and ‘manly’, the product of authentic emotional experience, not social privilege or polite manners. Asking what kind of character defined men like Gregory, Bell replied:

It is the factitious character that opens the way to public employment and professional honours: In what does it consist? in suavity of manners, a specious carriage, an agreeable person, a pleasing address, a facetious conversation, a thorough knowledge of the politics and courtliness of high life. A splendid establishment, a gaudy carriage, family connections, and the solicitation of friends . . .

Recalling Gregory's allusion to the ambulatory surgeon, Bell continued:

And will the Memorialist, a philosopher, and a liberal one, speak of these as specific qualities, which ascertain a man's professional skill? We hope, for the credit of bare unsophisticated nature, that the honest and feeling heart, the thinking head, and the steady hand! the open liberal hand, which drops its alms while it is assuaging pain! is not more frequent in the gilded chariot, than in the humble walks of life; where men drag along the burden of their duties, and crawl even on their lower extremities in the pristine manner.^96^

## V

Looking back on the conflict that had convulsed the Edinburgh medical community in the summer of 1800, Henry Cockburn claimed that Bell ‘had the best both of the argument and of the clever writing; but the public sided with the best laughter; and so Gregory was generally held to have the victory’.^97^ Certainly, whatever assessment might be made of their relative literary merits, there is little doubt that Bell lost the war. While his initial intervention had seemingly turned the opinion of the managers in the surgeons’ favour, a subsequent meeting in November 1800 altered the mode of attendance to one of six permanent appointees.^98^ Pointedly, while Bell was excluded, one of the appointments was his arch‐rival John Thomson. To add insult to injury, Thomson subsequently scooped two other prestigious offices; in 1804 he was appointed Chair of Surgery to the Royal College of Surgeons of Edinburgh, and in 1806 he was also awarded the newly created title of Regius Professor of Military Surgery at the university, despite the fact that it was Bell who had agitated for the creation of this post in the first place.^99^ Indeed, it has been said that Bell ‘never . . . quite recovered from his exclusion from the Infirmary’. Assisted by their brother, George Bell (1770–1843), John and Charles launched legal proceedings against the Royal Infirmary but were ultimately unsuccessful in getting the resolution overturned.^100^ In any case, John had given up teaching in 1800, devoting himself thenceforth to his private practice and to writing.

However, while Bell's career was frustrated by his dispute with Gregory, in another sense his reputation and influence were considerably enhanced. He continued to be a significant intellectual presence in Edinburgh surgery. For example, in 1808 Thomson trialled what he claimed was a new method of cutting for the stone at the Royal Infirmary. The technique was unsuccessful and led, in at least one case, to a patient's death. Bell published a scathing attack on the operative skill of a man who ‘after a life of busy, restless intrigue, in collusion with you [Gregory] and your worst associates, had procured himself to be elected surgeon to the Infirmary’.^101^ As a result, Thomson resigned his post at the Infirmary. According to his son, William Thomson (1802–52), he might normally have ‘treated the attack . . . with silent contempt . . . That he followed different procedure, is probably to be accounted for by the influence which he thought the statements of Mr Bell might exercise on the public mind.’^102^ By all accounts, Thomson had a very high regard for Bell's abilities as a surgeon, and rather less confidence in his own, something indicated by the fact that he was, at that very moment, seeking to redefine himself as a physician.^103^

Bell's posthumous reputation is evident in the surgeon John Struthers's mid‐nineteenth‐century account of the ‘Edinburgh Anatomical School’ in which he claimed that John Bell was ‘the reformer of Surgery in Edinburgh, or rather the father of it’. Moreover, Bell's legacy stretched far beyond the walls of the Old Town. During the period of his dispute with Gregory, which lasted from 1800 until around 1810, he published some of his most influential works. The most celebrated of these was his *Principles of Surgery* (1801–6). An ‘undying book’, in Struthers’ words, it outlined a comprehensive system of surgical disease and practice with a particular emphasis upon the *science* of surgery.^104^ What was also notable about the book was its concern with surgical *character* and *identity*. The first volume opened with a preliminary discourse on the ‘education and duties of a surgeon’ in which Bell not only emphasized the indispensability of a thorough anatomical education, but also advised that:

When a man enters upon such duties as these, he should be diligent, watchful, and laborious; humane, friendly, and self‐denying; full of unceasing anxiety for others, and a noble disregard of himself. We will not define the chief qualities of a surgeon, in this cold‐hearted way, of personal and showy accomplishments; mercy and tenderness towards his patients, and every kind of charity, are the chief virtues and most becoming ornaments of a surgeon.^105^

As can be seen, then, the emotional qualities of the surgeon continued to be one of the most characteristic features of Bell's writing and he expanded considerably upon these observations in 1810 with a book that, despite the intervening years, was explicitly addressed to James Gregory and whose title, *Letters on Professional Character and Manners: On The Education of a Surgeon, and the Duties and Qualifications of a Physician*, consciously referenced his father's *Lectures on the Duties and Qualifications of a Physician* (1772). In this text, Bell presented his most well‐rounded vision of the surgeon as man of sensibility: as compassionate, sympathetic and selfless, always placing his patient's interests before his own concern for reputation or fame. He was particularly concerned to deprecate the notion that operative flair, courage or daring should be regarded as admirable traits of surgical identity, arguing instead for an unostentatious and scientific character characterized by a dignified and considered deportment:

A man of science never proceeds without due reflection: The whole plan of his operation is perfect in his own mind: He commutes with his assistant rather by signs than words, and his manner commands that stillness which is due to a moment of suffering, and essential to his self‐possession and success: He is formed by education, and qualified, from the first moment in which he takes those public duties upon him, to give impressive lessons to the younger members of the profession: They are awe‐struck with the first horrors of incisions and blood, but depart with gratified feelings, when they see the scene closed with entire relief to the sufferer, and happy prospect of success; and they learn to love and respect their profession, and to study it with emulation.^106^

Bell's vision of the surgeon as calm, compassionate and composed would resonate across the succeeding decades. His *Principles of Surgery* was dedicated to the surgeons of London, including John Abernethy and Astley Cooper, and it is clear that he was as admired as much in England's capital as he was in Scotland's. Moreover, while it would be extravagant to claim that he was the inventor of what we might call the ‘Romantic model’ of the operative surgeon, a model in which Cooper and Abernethy were, in their own ways, equally formed, he was certainly its most passionate and articulate advocate. And this model, with its rejection of rashness and cruelty and its embrace of selflessness and compassion, can be said to have opened the door, rhetorically speaking, to surgery's transition from a craft to a modern scientific profession.

## Footnotes

1 James Gregory, *Additional Memorial to the Managers of the Royal Infirmary* (Edinburgh, 1803), p. 310.

2 Robert Chisholm, *The Life of Robert Chisholm, Bart*, I: *Autobiography* (Edinburgh, 1885), p. 86; Agnes Grainger Stewart, *The Academic Gregories* (Edinburgh, 1901), p. 136.

3 Henry Cockburn, *Memorials of his Time* (New York, 1856), p. 106.

4 Paul Lawrence, ‘Gregory, James (1753–1821)’, *Oxford Dictionary of National Biography* (Oxford, 2004) [hereafter: *ODNB*] <https://doi.org/10.1093/ref:odnb/11466> [accessed 14 Oct. 2018]; Royal College of Physicians of Edinburgh, *Narrative of the Conduct of James Gregory towards the Royal College of Physicians of Edinburgh* (Edinburgh, 1809).

5 For a biographical account see E. W. Wallis, ‘John Bell, 1763–1820’, *Medical History*, 8/1 (1964), pp. 63–9; K. Grudzien Baston, ‘Bell, John (1763–1820), *ODNB* <https://doi.org/10.1093/ref:odnb/2013> [accessed 14 Oct. 2018]; M. H. Kaufman, ‘John Bell (1763–1820), the “father” of surgical anatomy’, *Journal of Medical Biography*, 13/2 (2005), pp. 73–81.

6 For recent references to John's dispute with Gregory in relation to his brother, see Carin Berkowitz, *Charles Bell and the Anatomy of Reform* (Chicago, 2015), pp. 5–6, and Michael J. Aminoff, *Sir Charles Bell: His Life, Art, Neurological Concepts and Controversial Legacy* (Oxford, 2017), pp. 8–10.

7 Christopher Lawrence, ‘The Edinburgh Medical School and the end of the “Old Thing” 1790–1830’, *History of Universities*, 1 (1988), pp. 259–86, at p. 267, n. 42; L. S. Jaycna, ‘Robert Carswell and William Thomson at the Hôtel Dieu of Lyons: Scottish views of French medicine’, in Roger French and Andrew Wear (eds), *British Medicine in an Age of Reform* (London, 1991), pp. 110–34, at p, 116, n. 19; idem, *Philosophic Whigs: Medicine, Science and Citizenship in Edinburgh 1789–1848* (London, 1994), p. 56, n. 29; Guenter Risse, *New Medical Challenges During the Scottish Enlightenment* (Amsterdam, 2005), p. 51, n. 168.

8 Lawrence, ‘Edinburgh Medical School’, p. 267.

9 Risse, *New Medical Challenges*, pp. 50–1.

10 Michael Brown, ‘Medicine, reform and the “end” of charity in early nineteenth‐century England’, *English Historical Review*, 124/511 (2009), pp. 1353–88.

11 Gregory, *Additional Memorial*, pp. 102, 303.

12 John Struthers, *Historical Sketch of the Edinburgh Anatomical School* (Edinburgh, 1867), p. 41.

13 Cockburn, *Memorials of his Time*, p. 106.

14 For example, see James Wardrop, ‘Lectures on surgery’, *The Lancet*, 20/514 (6 July 1833), pp. 453–9, at p. 454.

15 Thomas Dixon, ‘“Emotion”: the history of a keyword in crisis’, *Emotion Review*, 4/338 (2012), pp. 338–44, at p. 340; see also idem, *From Passions to Emotions: The Construction of a Secular Psychological Category* (Cambridge, 2003).

16 Dixon, ‘“Emotion”’, p. 341.

17 Wallis, ‘John Bell’, p. 68; Baston, ‘Bell, John’.

18 For example, see Rob Boddice, *The Science of Sympathy: Morality, Evolution, and Victorian Civilization* (Urbana, IL, 2016).

19 On the politics of emotion, see William M. Reddy, *The Navigation of Feeling: A Framework for the History of Emotions* (Cambridge, 2001). On the politics of sensibility, see G. J. Barkar‐Benfield, *The Culture of Sensibility: Sex and Society in Eighteenth‐Century Britain* (Chicago, 1992); Markman Ellis, *The Politics of Sensibility: Race, Gender and Commerce in the Sentimental Novel* (Cambridge, 1996).

20 Ellis, *Politics of Sensibility*, pp. 220–1.

21 Dixon, ‘“Emotion”’, p. 341.

22 Lawrence, ‘Edinburgh Medical School’; Jacyna, *Philosophic Whigs*.

23 Christopher Lawrence, ‘Medicine as culture: Edinburgh and the Scottish Enlightenment’, PhD, University of London (1984), p. 27.

24 Ibid., pp. 314–21.

25 Ibid., pp. 103–11; Guenter Risse, *Hospital Life in Enlightenment Scotland: Care and Teaching at the Royal Hospital of Edinburgh* (Cambridge, 1986), pp. 25–33.

26 Quoted in Helen M. Dingwall, *‘A Famous and Flourishing Society’: The History of the Royal College of Surgeons of Edinburgh, 1505–2005* (Edinburgh, 2005), p. 98. Emphasis added.

27 Lawrence, ‘Medicine as culture’, pp. 111–17; Dingwall, *Famous and Flourishing*, pp. 97–9.

28 Quoted in Lawrence, ‘Medicine as culture’, p. 115.

29 See Marcus Ackroyd, et al., *Advancing with the Army: Medicine, the Professions, and Social Mobility in the British Isles 1790–1850* (Oxford, 2006); Catherine Kelly, *War and the Militarization of British Army Medicine, 1793–1830* (London, 2011).

30 Lawrence, ‘Edinburgh Medical School’, pp. 265–7.

31 Berkowitz, *Charles Bell*.

32 Struthers, *Historical Sketch*, p. 41.

33 Lawrence, ‘Edinburgh Medical School’, p. 267.

34 John Bell, *Letters on Professional Character and Manners: On the Education of a Surgeon, and the Duties and Qualifications of a Physician: Addressed to James Gregory, M.D. Professor of the Practice of Medicine in the University of Edinburgh* (Edinburgh, 1810), p. 548.

35 James Gregory*, Memorial to the Managers of the Royal Infirmary* (Edinburgh, 1800), p. 248.

36 M. H. Kaufman, ‘The excoriation of Benjamin Bell: who was Jonathan Dawplucker?’, *Journal of the Royal College of Physicians of Edinburgh*, 41 (2011), pp. 356–64.

37 Bell, *Letters on Professional Character*, p. 503.

38 Jonathan Dawplucker [John Bell], *Number Second, Being Remarks on the First Volume of Mr Benjamin Bell's System of Surgery* (1799).

39 Dawplucker [Bell], *Number Second*, pp. 53–5.

40 Henry Cockburn, *Journal of Henry Cockburn: Being a Continuation of the Memorials of his Time, 1831–1854*, II (Edinburgh, 1874), p. 203.

41 Lawrence, ‘Edinburgh Medical School’, p. 261.

42 Jacyna, *Philosophic Whigs*, pp. 2–5.

43 Berkowitz, *Charles Bell*, pp. 14–15, citing Adrian Desmond, *The Politics of Evolution: Morphology, Medicine, and Reform in Radical London* (Chicago, 1989), p. 93.

44 For an account of William's conversion, see Charles Bell, *Letters of Charles Bell* (London, 1870), pp. 4–7.

45
*Caledonian Mercury*, 2 July 1801, p. 3.

46 Gregory, *Additional Memorial*, pp. 80, 101.

47 Lawrence, ‘Edinburgh Medical School’, p. 268; Colin Kidd, ‘Burns and politics’, in Gerrard Carruthers (ed.), *The Edinburgh Companion to Robert Burns* (Edinburgh, 2009), pp. 61–73.

48 Paul Lawrence, Gregory [Gregorie], John (1724–1733), *ODNB* <https://doi.org/10.1093/ref:odnb/11457> [accessed 14 Oct. 2018].

49 Lawrence, ‘Gregory, James’.

50 Roger L. Emerson, *Academic Patronage in the Scottish Enlightenment: Glasgow, Edinburgh and St Andrews Universities* (Edinburgh, 2008).

51 Risse, *Hospital Life*, pp. 64–5.

52 Benjamin Bell, *Observations on the Mode of Attendance of the Surgeons of Edinburgh on the Royal Infirmary* (Edinburgh, 1800); John Thomson, *Outline of a Plan for the Regulation of the Surgical Department of the Royal Infirmary* (Edinburgh, 1800).

53 Gregory, *Additional Memorial*, p. 32.

54 Gregory, *Memorial*, p. 179.

55 Ibid., p. 3.

56 Ibid., pp. 25, 16, 18.

57 Ibid., p. 43.

58 Ibid., pp. 19–22.

59 Ibid., p. 35.

60 Ibid., p. 60.

61 Ibid., pp. 12, 63–4.

62 Ibid., pp. 68–9.

63 Brown, ‘Medicine, reform and the “end” of charity’.

64 Gregory, *Memorial*, p. 73.

65 Ibid., p. 99.

66 Ibid., pp. 74–5.

67 Ibid., pp. 78, 80–1.

68 Ibid., p. 204.

69 Ibid., p. 116.

70 Gregory, *Additional Memorial*, p. 137.

71 Gregory, *Memorial*, p. 15.

72 John Bell, *Answer for the Junior Members of the Royal College of Surgeons of Edinburgh to the Memorial of Dr James Gregory* (Edinburgh, 1800), p. i; Gregory*, Additional Memorial*, pp. 34–7.

73 Bell, *Answer*, pp. 1–2.

74 Ibid., p. 2.

75 Ibid., sect. II, pp. 6–7.

76 Michael Brown, ‘Surgery and emotion: the era before anaesthesia’, in Thomas Schilch (ed.), *The Palgrave Handbook of the History of Surgery* (London, 2017), pp. 327–48, at p. 338.

77 Bell, *Answer*, sect. II, p. 7.

78 Ibid.

79 Alan Mackintosh, *The Patent Medicines Trade in Georgian England: Constructing the Market by the Potency of Print* (London, 2018), pp. 52–4.

80 Ellis, *Politics of Sensibility*, pp. 1–9.

81 Christopher Lawrence, ‘The nervous system and society in the Scottish Enlightenment’, in Barry Barnes and Stephen Shapin (eds), *Natural Order: Historical Studies of Scientific Culture* (London, 1979), pp. 19–40; idem, ‘Medicine as culture’, pp. 133–87.

82 H. W. Drescher, ‘Mackenzie, Henry (1741–1831)’, *ODNB* < https://doi.org/10.1093/ref:odnb/17586> [accessed 14 Oct. 2018].

83 Ellis, *Politics of Sensibility*, p. 19; Maureen Harkin, ‘Mackenzie's “man of feeling”: embalming sensibility’, *English Literary History*, 61/2 (1994), pp. 317–40; Ildiko Csengei, ‘“I will not weep”: reading through the tears of Henry Mackenzie's “man of feeling”’, *The Modern Language Review*, 103 (2008), pp. 952–68.

84 Ellis, *Politics of Sensibility*, p. 35.

85 Ibid., ch. 6; Reddy, *Navigation of Feeling*, ch. 6; Thomas Dixon, *Weeping Britannia: Portrait of a Nation in Tears* (Oxford, 2015), ch. 8.

86 Ellis, *Politics of Sensibility*, p. 198.

87 Lionel Trilling, *Sincerity and Authenticity* (London, 1972); Tim Miles and Kerry Sinanan (eds), *Romanticism, Sincerity and Authenticity* (Basingstoke, 2010).

88 John Bell, *Discourses on the Nature and Cure of Wounds* (Edinburgh, 1795), pp. 74–5.

89 Bell, *Answer*, p. 5.

90 Ibid., p. 5.

91 Gregory, *Additional Memorial*, p. 281.

92 Bell, *Letters on Professional Character*, pp. 147–8.

93 Ibid., p. 543.

94 Michael Brown, *Performing Medicine: Medical Culture and Identity in Provincial England, c.1760–1850* (Manchester, 2011); idem, ‘Medicine, reform and the “end” of charity’.

95 Bell, *Letters on Professional Character*, p. 558.

96 Bell, *Answer*, sect. II, pp. 20–1.

97 Cockburn, *Memorials of his Time*, p. 106.

98 Dingwall, *Famous and Flourishing*, p. 106.

99 Jacyna, *Philosophic Whigs*, ch. 3; M. H. Kaufman*, The Regius Chair of Military Surgery in the University of Edinburgh, 1806–55* (Amsterdam, 2003), pp. 47–66.

100 Dingwall, *Famous and Flourishing*, p. 106.

101 Bell, *Letters on Professional Character*, p. 588.

102 William Thomson, ‘Biographical notice of Dr Thomson’, in J. Thomson, *An Account of the Life, Lectures, and Writings of William Cullen, M.D*., I (Edinburgh, 1859), pp. 5–82, at p. 33.

103 Jacyna, *Philosophic Whigs*, p. 98.

104 Struthers, *Historical Sketch*, p. 43.

105 John Bell, *The Principles of Surgery* (Edinburgh, 1801), p. 14.

106 Ibid., p. 559. Such lack of ostentation has also been observed in Bell's surgical illustrations. See Martin Kemp, ‘Style and non‐style in anatomical illustration: from Renaissance humanism to Henry Gray’, *Journal of Anatomy*, 216/2 (2010), pp. 192–208.

